# Up-regulation of bone marrow stromal protein 2 (BST2) in breast cancer with bone metastasis

**DOI:** 10.1186/1471-2407-9-102

**Published:** 2009-04-01

**Authors:** Dongqing Cai, Jie Cao, Zhen Li, Xin Zheng, Yao Yao, Wanglin Li, Ziqiang Yuan

**Affiliations:** 1 Key Laboratory for Regenerative Medicine of Ministry of Education, Ji Nan University, Guangzhou, PR China; 2 Joint Laboratory for Regenerative Medicine, The Chinese University of Hong Kong-Ji Nan University, Guangzhou, PR China; 3 Department of Surgery, Affiliated Guangzhou First People's Hospital, Guangzhou Medical College, Guangzhou, PR China; 4 Department of Molecular Genetics, Albert Einstein College of Medicine, New York, USA

## Abstract

**Background:**

Bone metastases are frequent complications of breast cancer. Recent literature implicates multiple chemokines in the formation of bone metastases in breast cancer. However, the molecular mechanism of metastatic bone disease in breast cancer remains unknown. We have recently made the novel observation of the BST2 protein expression in human breast cancer cell lines. The purpose of our present study is to investigate the expression and the role of BST2 in bone metastatic breast cancer.

**Methods:**

cDNA microarray analysis was used to compare the BST2 gene expression between a metastatic to bone human breast cancer cell line (MDA-231BO) and a primary human breast cancer cell line (MDA-231). The BST2 expression in one bone metastatic breast cancer and seven non-bone metastatic breast cancer cell lines were also determined using real-time RT-PCR and Western blot assays. We then employed tissue array to further study the BST2 expression in human breast cancer using array slides containing 20 independent breast cancer tumors that formed metastatic bone lesions, 30 non-metastasis-forming breast cancer tumors, and 8 normal breast tissues. In order to test the feasibility of utilizing BST2 as a serum marker for the presence of bone metastasis in breast cancer, we had measured the BST2 expression levels in human serums by using ELISA on 43 breast cancer patients with bone metastasis, 43 breast cancer patients without bone metastasis, and 14 normal healthy controls. The relationship between cell migration and proliferation and BST2 expression was also studied in a human breast recombinant model system using migration and FACS analysis.

**Results:**

The microarray demonstrated over expression of the BST2 gene in the bone metastatic breast cancer cell line (MDA-231BO) compared to the primary human breast cancer cell line (MDA-231). The expression of the BST2 gene was significantly increased in the bone metastatic breast cancer cell lines and tumor tissues compared to non-bone metastatic breast cancer cell lines and tumor tissues by real time RT-PCR, Western blot and TMA. Furthermore, serum levels of BST2 measured by ELISA were also significantly higher among patients with breast cancer metastatic to bone compared to breast cancer patients without metastatic to bone (P < .0001). Most importantly, the breast cancer cell line that transfected with BST2 demonstrated increased BST2 expressions, which was associated with increased cancer cell migration and cell proliferation.

**Conclusion:**

These results provide novel data indicating the BST2 protein expression is associated with the formation of bone metastases in human breast cancer. We believe that BST2 may be a potential biomarker in breast cancer with bone metastasis.

## Background

Breast cancer remains a major public health challenge in the United States, with approximately 215,990 new cases among women and 40,000 deaths projected for 2006 [1]. Bone metastasis is the most common complication of breast cancer and skeletal involvement is present in 70% of breast cancer autopsy cases [2]. Therefore, early detection of bone metastases will improve the quality of life and decrease morbidity and mortality [1]. Studies are currently in progress to look at ways to prevent metastatic breast cancer in women. Multiple literatures have reported that abnormal elevation rates of biomarkers, 34.6% for carcinoembryonic antigen (CEA), 30.8% for carbohydrate antigen 19-9 (CA19-9) and 30.8% for cancer antigen 125 (CA125) have been observed in cases of bone metastasis [3]. However, there is no sensitive, specific and low-cost test to detect early bone metastases [4]. In order to discover a sensitive and specific biomarker for detection of early bone metastases in breast cancer, we have analyzed gene expressions of MDA-231BO, a bone metastatic breast cancer cell line and compared it to MDA-231, a non-bone metastatic breast cancer cell line by cDNA microarray. In our present study, we present a novel analysis of differential expressions of bone marrow stromal protein 2 (BST2) in the breast cancer with bone metastasis vs. breast cancer without bone metastasis.

The BST2 gene is also known as the HM1.24 antigen located on chromosome 19p13.2 [5]. It is a transmembrane glycoprotein with a molecular weight of 35 kDa and consists of 180 amino acids [6]. BST2 is expressed on mature, normal and neoplastic B cells, but not on other cells in the peripheral blood, bone marrow, liver, spleen, and breast tissues of healthy individuals or patients with plasma-cell malignancies [6]. The BST2 was previously reported that it may be involved in pre-B cell growth via cell-cell interaction by Dr Ohtomo [7]. The BST2 expression has been identified in multiple myeloma and involved in the tumor invasion and progression [8,9]. Walter-Yohrling reported that higher levels of BST2 were observed in metastatic ovarian cancer tissues than non-metastatic ovarian cancer tissues [9]. In our present study, we investigated the expression and the role of BST2 in the initiation and development of bone metastatic breast cancer.

The process of bone metastasis is believed to occur in three steps: "(1) proliferation and invasion of cancer cells at a primary site, (2) intravasation, migration in the circulation and extravasation of cancer cells, and (3) Specific for bone metastases, the arrest of cancer cells in the bone marrow, egress from central sinus, attachment to bone surfaces, osteoclastic bone destruction, and colonization in bone" [10,11]. BST2 is a 35-kDa membrane protein characterized by a tandem repeat of three cis-elements in the promoter region for a transcription factor, signal transducer and activators of transcription 3 (STAT3), which ultimately mediates the IL-6 response gene expression [12]. It is well-known that STAT3 and to some extent STAT1 is activated in breast cancers and that constitutively active mutants of STAT3 promote the growth and survival of tumor cells thereby contributing to malignancy [13-15]. STAT3 and STAT1 can be activated by various receptors and non-receptor tyrosine kinases [14,16]. It is conceivable that BST2 is an important regulator in the STAT3/BST2/IL6 pathway leading to increased cell proliferation, as well as osteoclastic bone destruction and/or production in the bone metastatic breast cancer. In our present study, we employed RNAi techniques to suppress the BST2 expression to investigate the effects of BST2 modulation on breast cancer cell proliferation, migration and invasion.

## Methods

### Cell lines

MDA-231, a non-bone metastatic breast cancer and MDA-231BO, bone metastatic breast cancer cell lines for cDNA microarray assay were obtained from Dr. Yoneda. Additionally, a normal breast cell line (MCF-10A) and six primary breast cancer cell lines (HTB-121, BC701, UACC812, MCF-7, T47D and MDA-468) were purchased from the American Type Culture Collection (ATCC, Inc). The cell lines were maintained in Dulbecco's modified Eagle's medium (DMEM) with L-glutamine supplemented with 10% fetal bovine serum, 50 units/ml Streptomycin-penicillin, 1% non-essential amino acid, and HPEBS. They were incubated at 37 degrees C with 5% CO_2_. The RNAs, DNAs and proteins from all the cell lines were isolated with Trizol (Life Technologies, Carlsbad, CA) according to kit instructions.

### cDNA Microarray analysis

A bone-seeking clone (MDA-231BO) of the human breast cancer cell line MDA-231 was constructed by sequential passages in mice and the metastatic cells collected from bone [17]. The biological characteristics of MDA-231BO were identified with higher occurrences in metastasis to the bone compared with the MDA-231 parental cells (MDA-231 cell line) [17]. In order to discover a sensitive and specific biomarker for detection of early bone metastases in breast cancer, we analyzed the differential gene expressions of MDA-231BO, a bone metastatic breast cancer cell line and compared it to MDA-231, a non-bone metastatic breast cancer cell line. The total RNA was extracted from MDA-231BO and MDA-231 cell lines, as previously described [18]. The total RNA (100 ug) was used to produce labeled cDNA by anchored oligo (dT)-primer reverse transcription using SuperScript II reverse transcriptase (Life Technologies, Inc, Carlsbad, CA) in the presence of fluorescent dye, Cy5-dUTP or Cy3-dUTP (Amersham, Piscataway, NJ), respectively. The robot in the cDNA microarray facility at the Albert Einstein College of Medicine (AECOM) has the precision to spot 9568 PCR products onto a single glass slide. From Genome Systems, we have obtained 9568 unique human cDNAs from the I.M.A.G.E. consortium that represent 15–20% of all human genes [19] (AECOM website: http://microarray1k.aecom.yu.edu). The fluorescent cDNA probes were then hybridized to Silane glass slides with 9568 cDNA human gene spots were hybridized according to AECOM standard protocol in which each slide was probed with bone metastatic breast cancer and non-bone metastatic breast cancer. Slides were scanned in our microarray facility scanner. Data from the hybridization reactions are collected using a two-colored laser scanning confocal microscope that is custom designed and built at AECOM specifically for maximum sensitivity necessary to measure low abundance mRNAs. The images were exported to the GenePix Pro 3.0 software (Axon Instruments, Inc, Union City, CA) for signal intensity analysis. Poor-quality spots were flagged and excluded from analysis. Signal intensity information was exported to Excel. The data was normalized for statistical analysis. Locally weighted linear regression (LOWESS) analysis is a widely used normalization method to reduce systematic errors in the measured expression levels. Normalization algorithms can be applied either globally (to the entire data set) or locally (to some physical subset of the data) by GenePix software. The **output **file of normalized data can be saved directly to your hard drive, and can be used as the input file for the "Filtering" module. The "Normalization" module also generates a graphical file containing data plots of Ratio vs. Geometrical Mean of R&G Intensity (before and after normalization). This module performs data filtering by eliminating bad/absent flag spots and weak signals (lower than "A" cutoff). The **input file **was the result file from the "Normalization" module. The **output **file can be saved directly to your hard drive, and can be used as an input file for the "Comparison" module. This module performs Ratio filtering. For one dataset (chip), this module performs Ratio filtering (Up & Down regulated genes). When comparing multiple datasets, the Ratio filtering test must be satisfied in all datasets for a given gene to be selected. Testing is satisfied by expression ratios in either the same or opposite direction. The **input file **was the result file from the "Filtering" module. Data were sorted based on "fold change," and genes with at least 1.5-fold up-regulation or down-regulation were accepted as significant alteration and the genes have a significant differential expression in at least four of five experiments chosen for further consideration.

### Measurement of BST2 mRNA and protein expressions in breast cancer cell lines by Real time RT-PCR and Western Blot Assays

Total RNAs and proteins were isolated from each cell line (MCF-10A, HTB-121, UACC812, MCF-7, T47D, MDA-468, BC701, MDA-231 and MDA-231BO) as previously described [18] and BST2 mRNA and protein expressions determined by real time RT-PCR and Western blot assays. Commercial BST2-P polyoclonal antibody against BST2 was optimized for Western blot assay (FabGennix Inc, Texas). For each sample, the expression of the BST2 gene and the β-actin loading control was determined. The ratio of the target-to-β-actin was calculated as the normalized value. The real time PCR and Western blot were repeated three times.

### Delineation of BST2 expression in human breast cancer tissueusing Tissue Microarray-Immunohisochemistry (TMA-IHC)

BST2 expression in human breast tissue specimens (normal breast, non-bone metastatic breast cancer and bone metastatic breast cancer tissues) was assessed using TMAs stained with BST2 antibody. Commercial BST2-P polyoclonal antibody against BST2 was optimized for TMA-IHC assay (FabGennix Inc, Texas, USA). TMAs were purchased from MTR Scientific (MTR Scientific Inc, MD) and were comprised of 116 tissue cores from 50 breast tumors (30 non-bone metastatic and 20 bone metastatic breast tumors) and 8 normal breast samples. These 20 specimens were from the bone metastasis group of the patients with bone metastasis only. Breast cancer tissues were collected from 50 breast cancer patients with and without clinical and bone scan evidences of bone metastases prior to treatment. The bone metastasis and non-bone metastasis were identified by bone scan examination. The slides were de-paraffinized with xylene and rehydrated with decreasing concentrations of alcohol and water. Heat-induced epitope retrieval was performed by placing slides in plastic coplin jars and a citrate buffer (pH 6.0; BioGenex, Sandown, NH) using a decloaking chamber (Biocare Medical, Walnut Creek, CA) for 30 minutes. After heat-induced epitope retrieval, endogenous peroxidase was blocked with 0.3% H_2_O_2 _in PBS for 20 minutes, followed by washing twice in PBS and then incubated for 30 minutes with biotinylated secondary antibodies (Vector Laboratories) diluted to 1:250 with universal blocking reagent and then incubated for 45 minutes with an avidin-biotin complex method reagent (Vectastain Elite ABC kit; Vector Laboratories). After development, slides were washed twice with distilled water, lightly counterstained with Mayer's hematoxylin, dehydrated, cleared, and mounted with resinous mounting medium. This protocol has been approved by the IRB of Affiliated Guangzhou First People's Hospital, Guangzhou Medical College, and AECOM. Analysis of TMA-IHC included the following: A quantitative analysis of the immune reactive sections related to the total tissue area was performed. We measured the percentage of positive immune reactivity using a mouse anti-human BST2 Ab in the tissue epithelium (normal and tumor). The histological images were captured with an OLYMPUS BX50 system microscope (Olympus Microscopes, Tokyo, Japan) with an objective magnification of 20×, through a video camera (Sony, Tokyo, Japan) and digitized with Image-Pro Plus for Windows software. In each slide, 300 cells per core were measured.

### Delineation of BST2 serum levels in breast cancer cases by enzyme-linked immunoabsorbent assay (ELISA)

The peripheral serums were collected from 86 breast cancer patients with and without clinical and bone scan evidences of bone metastases prior to treatment. The bone metastasis and non-bone metastasis were identified by bone scan examination. BST2 expressions was measured in the serum from 14 normal healthy individuals, 43 non-bone metastatic breast cancer and 43 bone metastatic breast cancer cases using ELISA as per our standard protocol. The BST2-Fc antibody in 1 μg/ml PBS was added to 96-well plates respectively, and incubated for 1 hour at 37°C with serum samples. Reactivity was measured by absorbance at 450 nm. Resulting antigen levels were presented as a mean difference from OD at 450 nm of mAb coated wells. OD obtained without serum was subtracted from mean OD of the sample wells. The ELISA experiment was repeated three times. This protocol for human samples has been approved by the IRB.

### Effects of BST2 on migration of breast cancer cells were analyzed by migration assay

The migration assay was measured using Transwell (Costar, NY, pore size, 8-μm) in 24-well dishes. Before performing the migration assay, 0.5 and 3 μg of pcDNA3-BST2 or pcDNA3-empty vector was transfected into 1 × 10^6 ^MDA-231 cells, a human breast cancer cell line with low metastasis (BST2 deficient), respectively, using the PolyFect Transfection Reagent kit (Qiagen, CA). MDA-231^+pcDNA3-BST2 ^with BST2 proficiency and MDA-231^+empty vector ^with BST2 deficiency were suspended at 24 hours post-transfection. Approximately 2 × 10^4 ^cells in a 100 μl of RPLM-1640 medium without serum were placed in the upper chamber. In the lower chamber, 200 μl of osteoblast condition medium was placed. The plates were incubated for 24 hours at 37°C in 5% CO2. Then, the cells were fixed for 15 min in methanol and stained for 15 min with 0.05% crystal violet. Cells were counted under microscope. The expression level of BST2 protein also was detected using Western blot assays. β-actin was used as a loading control. Each clone was plated in triplicate in each experiment, and each experiment was repeated at least three times.

### Fluorescence-activated cell sorting (FACS) analysis of cell cycle and apoptosis

Cell cycle distribution and apoptosis were analyzed in MDA-231^+pcDNA3-BST2 ^and MDA-231^+empty vector ^breast cancer cell lines using a FACS apparatus (Becton-Dickinson, KY). The cells were gathered at 24, 36 and 48 hours post-transfection, washed with PBS, and centrifuged. Pellets were fixed with ice-cold 70% ethanol for 1 hour at 4°C. The cells were then centrifuged for 5 min, the pellets were washed, re-suspended in PBS, and treated with RNase A at 5 μg/mL at 37°C for 30 minutes. The cells were chilled over ice for 10 minutes and stained with Propidium iodide at 50 μg/mL for 30 min at room temperature in darkness. Subsequent analysis of cell-cycle distribution and apoptosis were performed by FACS using the FACScan (Becton-Dickinson, KY). Each of these experiments was repeated at least three times.

## Results

### Up-regulation of the BST2 gene in bone metastatic breast cancer cell line by cDNA microarray

**Figure 1:**
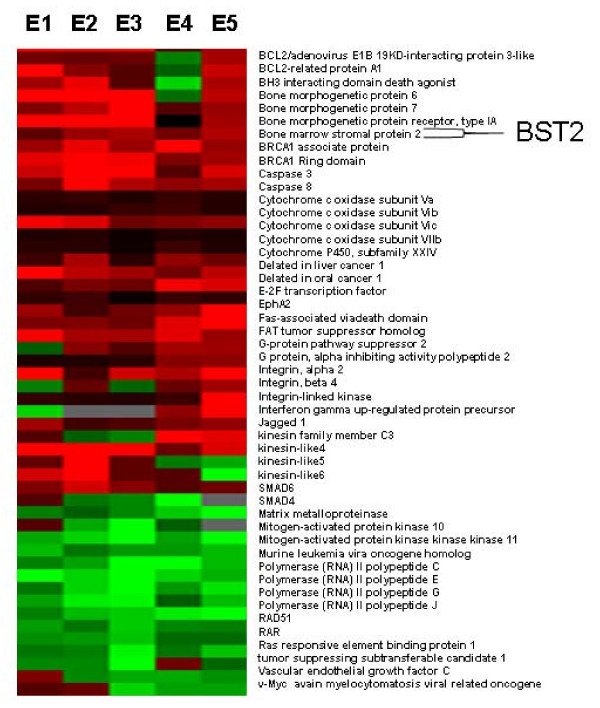
**Treeview of differential gene expression data from bone metastatic breast cancer cell line, MDA-231BO vs. primary breast cancer cell line, MDA-231)**. Treeview demonstrates the differential gene profiles in the bone metastatic breast cancer cell line compared to primary breast cancer cell line. Up-regulation is indicated by red, down-regulation by green, and no significant change by dark. Figure 1 shows 50 genes with greatest differential expression in five cDNA microarray experiments.

The cDNA microarray was performed as described above, comparing a bone metastatic breast cancer cell line (MDA-231BO) to a non-bone metastatic breast cancer cell line (MDA-231), which served as a reference. In all 5 cDNA microarray experiments, 124 poor-quality spots were flagged and excluded from our analysis. We identified 172 genes, including 20 expressed sequence tags (ESTs), with significant differential expression in at least four of the five experiments. From the structure and function of the genes, we selected several of interest for further study. We found BST2, an important bone marrow stromal protein, to be significantly up-regulated in the bone metastatic breast cancer cell line, MDA-231BO, compared to MDA-231 (Expression ratios = 3.2, 4.5, 4.29, 3.97 and 4.9) (Figure 1).

### Measurement of BST2 mRNA and protein expression in breast cancer cell lines by real time RT-PCR and Western Blot Assays

**Figure 2:**
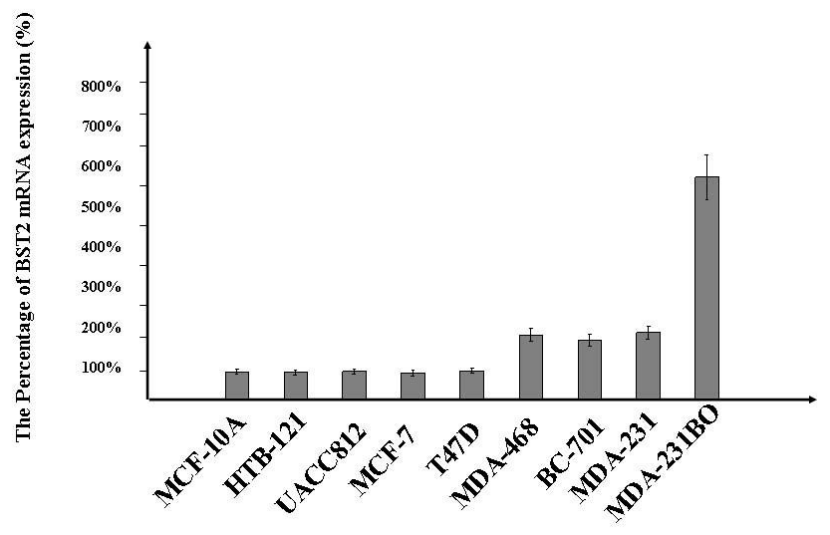
**Real time RT-PCR demonstrates increased expression of BST2 mRNA in bone metastatic breast cancer cell line: MDA-231BO compared to non-bone metastatic breast cancer cell lines: TB-121, UACC812, MCF-7, T47D, MDA-468, BC-701, MDA-231 and normal breast cell line: MCF-10A**. β-Actin was used to as a loading control.

**Figure 3:**
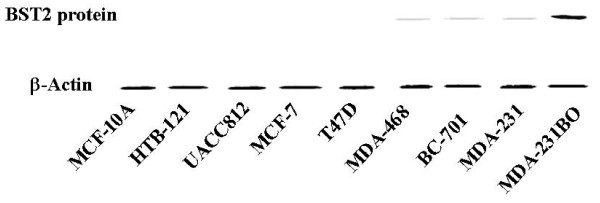
**Western blot demonstrates increased expression of the BST2 protein in bone metastatic breast cancer cell line: MDA-231BO compared to non-bone metastatic breast cancer cell lines: TB-121, UACC812, MCF-7, T47D, MDA-468, BC-701, MDA-231 and normal breast cell line: MCF-10A**. β-Actin was used to as a loading control.

Microarray results were confirmed and expanded by real time RT-PCR and Western blot analyses. The BST2 mRNA was significantly increased (P < 0.01) in the bone metastatic breast cancer cell lines, MDA-231BO compared to the normal breast cell line, MCF-10A, and the non-bone metastatic breast cancer cell lines, HTB-121, UACC812, MCF-7, T47D, MDA-468, BC-701 and MDA-231 by real time RT-PCR (Figure 2). The expression level of BST2 protein was also increased in bone metastatic breast cancer cell line, MB-231BO compared to the normal breast cell line, MCF-10A and non-bone metastatic breast cancer cell lines, HTB-121, UACC812, MCF-7, T47D, MDA-468, BC-701 and MDA-231 by Western blot assay (Figure 3).

### Delineation of BST2 expression in human breast cancer tissue using Tissue Microarray (TMA) Immunohisochemistry (IHC)

**Figure 4:**
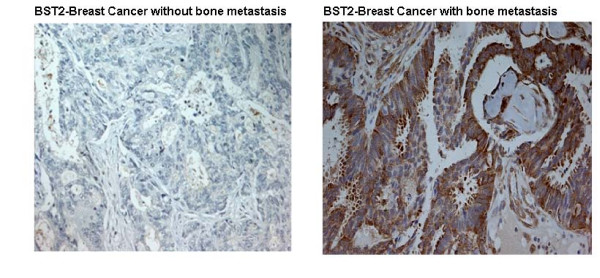
**This figure shows the BST2 expression in human breast cancer samples as determined by tissue array**. The bone metastatic breast cancer tissue (B) show the robust level of the BST2 expression, whereas the BST2 expression is absent in the non-bone metastatic breast cancer tissues (A). The brown staining in bone metastatic breast cancer cells demonstrates the significantly increased expression of BST2.

We characterized each of our 50 human breast tumor samples (present on our TMA). The tumor sample was classified as BST2 deficient as it was negatively stained (absence of brown indicates reduced expression). We observed that 1 out of 30 (3.3%) non-bone metastatic breast cancer tissues and 11 out of 20 (55%) bone metastatic breast cancer tissues showed increased BST2 expression, but none of the 8 (0.0%) normal breast tissues displayed the increased expression (Figure 4). Applying the one-tail Exact Fisher Test to this data, we obtained a p-value < 0.0001. Our results indicated that the BST2 expression was significantly increased in the bone metastatic breast cancer tissues.

### Delineation of serum levels of BST2 in breast cancer cases byELISA

To assess the sensitivity in ELISA, two-fold serial dilutions of recombinant BST2 proteins (1.25–120 pg/ml) were used. We demonstrated that minimum detection limits ranged from 1.25–5 pg/ml. Our result presented that the mean absorption value + SD for normal healthy individuals, Non-bone metastatic Group, Bone metastatic Group were 0.22 ± 0.05, 0.24 ± 0.11 and 0.51 ± 0.15, respectively (Table 1). To evaluate the specificity of BST2 expression in breast cancer with bone metastasis, 86 serum samples from patients previously diagnosed with and without bone metastasis by bone scan were determined by ELISA. In all 43 BMBC cases, we demonstrated that 36 (84%) of 43 had increased serum levels of BST2 (BST2 > 0.26). Among the control group, 3 (7%) of 43 NBMBC patients had increased serum levels of BST2 (BST2 > 0.26). Thus, both sensitivity and specificity of this assay were 84% (36/43) and 93% (40/43), respectively. Our results indicated that breast cancer patients with bone metastasis exhibited significantly increased serum BST2 as compared to breast cancer patients without bone metastasis as well as normal healthy individuals (p < .001) (Table 1).

**Table 1:** Serum BST2 level in normal healthy individuals, non-bone metastatic and bone metastatic breast cancer cases

	Serum levels					
	
	N	*> 0.26*	Mean Dev	Std	Min	Max
Normal Healthy Group	14	0 (0%)	0.22	0.05	009	0.26
Non-bone metastatic Group	43	3(7%)	0.24	0.11	0.17	0.52
Bone metastatic Group	43	36(84%)^a^	0.51^b^	0.15	0.22	0.81

^a^: P = 0.0006 (P < 0.001), ^b^: P = 0.02 (P < 0.05)

### Effect of BST2 on the migration ability of breast cancercells

**Figure 5:**
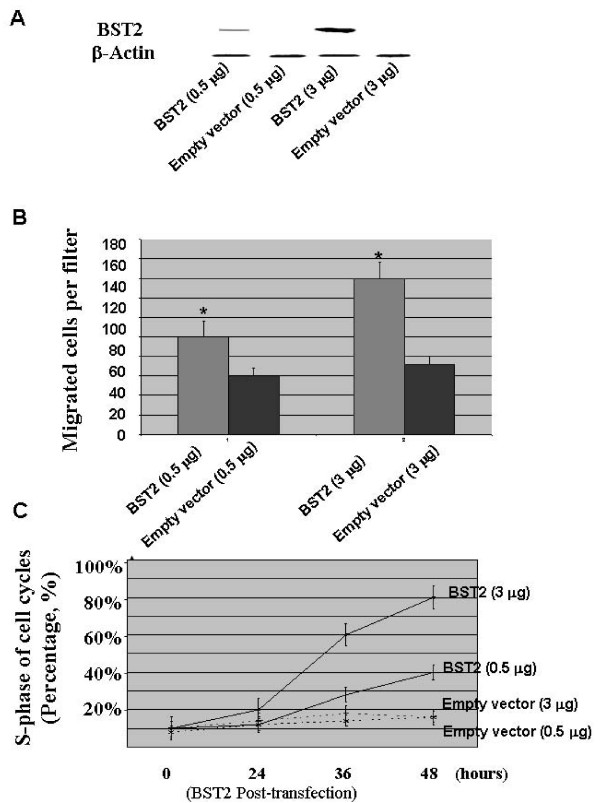
**A: Before performing the cell migration and proliferation assays, protein expression of BST2 were measured by Western blot in MDA-231 cell line after transfection at 24, 36 and 48 hours with pcDNA3-BST2 (0.5 and 3 μg) and pcDNA3 empty vector (0.5 and 3 μg), respectively**. Figure 5A shows that the BST2 protein expression is significantly increased by transfection with pcDNA3-BST2. β-Actin was used as a loading control. **B: **The graph shows that the migration activities of breast cancer cells (MDA-231) were measured at the 48-hour post-transfection with pcDNA3-BST2 (0.5 and 3 μg) and pcDNA3 empty vector (0.5 and 3 μg), respectively. Figure 5B indicates significantly increased the pcDNA3-BST2-treated cell line (MDA-231) compared to the cells treated with the empty vector (P < 0.01) (5B). **C: **The graph shows a FACS quantitative assessment of cells in the S phase of cell cycles in the MDA-231 cell line. Figure 5C represents the MDA-231 cell line at 24, 36 and 48-hour post-transfection with pcDNA3-BST2 (0.5 and 3 μg) and pcDNA3 empty vector (0.5 and 3 μg), respectively. The S-phase cell population significantly increased in the pcDNA3-BST2-treated cell line (MDA-231) compared to the cells treated with the empty vector (P < 0.01) (5C). These graphs show the mean values from three independent experiments.

The migration ability of BST2 on breast cancer cells was analyzed using an in-vitro migration assay. The BST2 expression was induced into MDA-231, a human breast cancer cell line with low BST2 expression (Figure 5A). At 48 hours post-transfection with BST2, the migration activities significantly increased in the pcDNA3-BST2-treated BST2 deficient cell line (MDA-231) compared to the cells treated with the empty vector (P < 0.01) (Figure 5B).

### Effect of BST2 on cell proliferation of breast cancer cells

To determine whether the BST2 expression contributes to cell proliferation and apoptosis profiles, we assessed cell cycle and apoptosis profiles by FACS in a recombinant BST2 cell model. At 24, 36 and 48 hours post-transfection with BST2, the S-phase cell population significantly increased in the pcDNA3-BST2-treated BST2 deficient cell line (MDA-231) compared to the cells treated with the empty vector (P < 0.01), (Figure 5C). However, the percentage of apoptotic cells was not significantly altered in the pcDNA3-BST2-treated BST2 deficient cell line (MDA-231) compared to the cells treated with the empty vector (P > 0.05).

## Discussion

Bone metastasis is one of the most common complications in patients with breast cancer. However, the precise mechanism of metastatic bone disease in breast cancer remains unknown. Recently, up-regulation of BST2 mRNA was reported in endometrial cancer [20]. However, the BST2 protein expressions aren't identified in the breast cancer. In our current study, we have identified BST2, an important bone marrow stromal cell growth factor, as significantly up-regulated in a BMBC, compared to a NBMBC by cDNA microarray (Figure 1). These results were further confirmed in a large number of breast cancer cell lines using real time RT-PCR and Western-blot assays (Figure 2 and 3). In a subsequent study, we expanded our investigation of BST2 expressions in human bone metastatic breast cancer and non-bone metastatic breast cancer using TMA-IHC. We demonstrated that the BST2 expression was significantly increased in human bone metastatic breast cancer tissues compared to human non-bone metastatic breast cancer and normal breast tissues by TMA (Figure 4). This important finding supports our initial cell-line-based results. In order to discover a novel, simple and sensitive biomarker in bone metastatic breast cancer, we also measured serum BST2 level using ELISA. Thirty-six (84%) of 43 breast cancer patients with bone metastasis had significantly increased serum levels of BST2 as indicated by ELISA (Table 1). Among the control group, 3 (7%) of 43 breast cancer patient without bone metastasis had significantly increased serum levels of BST2. The breast cancer patients with bone metastasis exhibited significantly increased serum BST2 as compared to breast cancer patients without bone metastasis and normal healthy individuals (p < .0001) (Table 1). These results indicated that a novel biomarker, BST2, is up-regulated in breast cancer cells with bone metastasis compared to in-breast cancer cells without bone metastasis.

Cell proliferation, migration and invasion of cancer cells at a primary site is an important step in the process of bone metastasis [10]. In this study, we determined the migration activities of breast cancer cells by an in-vitro migration assay. We induced BST2 expressions in a breast cancer cell line (MDA-231). Our results showed that the migration activities were significantly increased in the MDA-231 cells with high BST2 expression compared to the MDA-231 cells with low BST2 expression (P < 0.01) (Figure 5B). Additionally, we further assessed the role of BST2 expression in the regulation of cell cycles progression and apoptosis in breast cancer cells, and we induced BST2 expressions in a breast cancer cell line, MDA-231. Subsequent FACS analysis indicated increased S-phase fractions following induction of BST2 expressions (Figure 5C). We reassessed apoptosis activities in the MDA-231 cell line post-BST2 induction and observed insignificant alteration in apoptosis by FACS. The results suggest that the variable BST2 expression contributes to the alteration of cell cycle kinetics in human breast cells. BST2 is a 35-kDa membrane protein characterized by a tandem repeat of three cis-elements in the promoter region for a transcription factor, signal transducer and activators of transcription 3 (STAT3), which ultimately mediates the IL-6 response gene expression [12]. It is well-known that STAT3 and to some extent STAT1 is activated in breast cancers and that constitutively active mutants of STAT3 promotes the growth and survival of tumor cells thereby contributing to malignancy [13-15]. STAT3 and STAT1 can be activated by various receptor and non-receptor tyrosine kinases [14,16]. Interleukin-6 (LI-6) is an important growth and survival factor by the regulation of phosphatidylinositol 3-kinase signaling pathway in the initiation and progression of human cancers [21]. It is conceivable that BST2 may be an important regulator in the STAT3/BST2/IL6 pathway leading to increased cell migration and proliferation in the bone metastatic breast cancer.

## Conclusion

We conclude that we have demonstrated that the BST2 expression is significantly increased in bone metastatic breast cancer. We believe that BST2 may be a potential biomarker in bone metastatic breast cancer. We hope to show that it will provide a convenient and sensitive diagnostic method for breast cancer patients with bone metastasis and facilitate the development of novel diagnostic and therapeutic interventions designed to prevent bone metastases in breast cancer.

## Competing interests

The authors declare that they have no competing interests.

## Authors' contributions

DC, ZQY designed research and wrote the paper. ZQY performed the research (cDNA microarray). JC performed research (collected samples). ZL, performed research (Real-time PCR and Western Blot assays). XZ, performed research (cell culture, FACS and migration assays). YY performed research (BST2 transfection and IHC). WL performed research (ELISA assay).

## Pre-publication history

The pre-publication history for this paper can be accessed here:

http://www.biomedcentral.com/1471-2407/9/102/prepub
